# Robotic Esophagectomy. A Systematic Review with Meta-Analysis of Clinical Outcomes

**DOI:** 10.3390/jpm11070640

**Published:** 2021-07-06

**Authors:** Michele Manigrasso, Sara Vertaldi, Alessandra Marello, Stavros Athanasios Antoniou, Nader Kamal Francis, Giovanni Domenico De Palma, Marco Milone

**Affiliations:** 1 Department of Advanced Biomedical Sciences, University of Naples “Federico II”, Via Pansini 5, 80131 Naples, Italy; michele.manigrasso@unina.it; 2 Department of Clinical Medicine and Surgery, University of Naples “Federico II”, Via Pansini 5, 80131 Naples, Italy; vertaldisara@gmail.com (S.V.); alessandramarello@gmail.com (A.M.); giovanni.depalma@unina.it (G.D.D.P.); 3 Medical School, European University Cyprus, 2404 Nicosia, Cyprus; stavros.antoniou@hotmail.com; 4 Department of Surgery, Mediterranean Hospital of Cyprus, 3117 Limassol, Cyprus; 5 Yeovil District Hospital, Somerset BA21 4AT, UK; nader.francis@ydh.nhs.uk

**Keywords:** robotic, esophagectomy, esophageal cancer, laparoscopic, open surgery

## Abstract

**Background:** Robot-Assisted Minimally Invasive Esophagectomy is demonstrated to be related with a facilitation in thoracoscopic procedure. To give an update on the state of art of robotic esophagectomy for cancr a systematic review with meta-analysis has been performed. **Methods:** a search of the studies comparing robotic and laparoscopic or open esophagectomy was performed trough the medical libraries, with the search string “robotic and (oesophagus OR esophagus OR esophagectomy OR oesophagectomy)”. Outcomes were: postoperative complications rate (anastomotic leakage, bleeding, wound infection, pneumonia, recurrent laryngeal nerves paralysis, chylotorax, mortality), intraoperative outcomes (mean blood loss, operative time and conversion), oncologic outcomes (harvested nodes, R0 resection, recurrence) and recovery outcomes (length of hospital stay). **Results:** Robotic approach is superior to open surgery in terms of blood loss *p* = 0.001, wound infection rate, *p* = 0.002, pneumonia rate, *p* = 0.030 and mean number of harvested nodes, *p* < 0.0001 and R0 resection rate, *p* = 0.043. Similarly, robotic approach is superior to conventional laparoscopy in terms of mean number of harvested nodes, *p* = 0.001 pneumonia rate, *p* = 0.003. **Conclusions:** robotic surgery could be considered superior to both open surgery and conventional laparoscopy. These encouraging results should promote the diffusion of the robotic surgery, with the creation of randomized trials to overcome selection bias.

## 1. Introduction

Esophageal cancer represents the seventh most common cause of cancer morbidity and the sixth cause of cancer-related death [1].

Radical esophagectomy with lymphadenectomy represents nowadays the milestone for the treatment of esophageal cancer [2]. Since its introduction in the late 1940s, open esophagectomy has been adopted for a long time, obtaining considerable oncologic results [3]. In the new era of minimally invasive laparoscopic surgery, minimally invasive esophagectomy started to be performed in the 2000s, providing the well-known advantages on recovery of the minimally invasive procedures. Safety and efficacy of minimally invasive esophagectomy has been reported in several experiences [4,5,6,7], further providing similar oncologic results and long term recurrence rate [8,9,10]. However, on a clinical point of view, the introduction of minimally invasive esophagectomy in the clinical practice is far to be considered as a standard of care. Major reason for that should be considered technical challenges in performing minimally invasive esophagectomy.

Since its introduction in 2000s, robotic surgery has been adopted to overcome technical difficulties of laparoscopic surgery. The facilities of the robotic approach lay in the intrinsic characteristics of the robotic platforms. In fact, the three-dimensional view allowed a better visualization of the operative field and the EndoWrist^®^ technology with the seven-degrees movement of the robotic arms allows to perform more accurate movements in narrow space [11,12]. Even if robotic approach could be considered the gold standard only for the treatment of the prostate cancer, it has accumulated consensus in many surgical fields [13,14,15,16]. In the setting of minimally invasive esophagectomy, it was first introduced in 2003 by Kernstine et al. [17], but controversies about the advantages of robotic approach have to be considered still an open issue.

Interest about the results of robotic surgery, also in comparison with open and laparoscopic approach, is fervent worldwide. Results on robotic esophagectomy were accumulated exponentially in the last years providing advantages of robot-assisted surgery. 

To delineate the state of art of robotic approach to treat esophageal cancer, we have designed a systematic review and meta- analysis comparing robotic with both open and laparoscopic surgery, toward to the identification of a gold standard treatment. 

## 2. Materials and Methods

### 2.1. Literature Search and Study Selection

This systematic review complied with PRISMA (Preferred Reporting Items for Systematic reviews and Meta-Analyses) reporting standards [18] and was developed in line with Meta-Analysis of Observational Studies Epidemiology (MOOSE) guidelines [19].

Cochrane Library, EMBASE, PubMed, SCOPUS, and Web of Science were interrogated. The search string “robotic and (oesophagus OR esophagus OR esophagectomy OR oesophagectomy)” was used. Only articles published in English were considered.

Indexed abstract of posters and podium presentations at international meetings were not included. We did not consider systematic reviews and meta-analyses. However, the latter were consulted to identify additional studies of interest. The reference lists of retrieved studies were reviewed. In case of overlapping series in different studies, only the most recent article was included.

The research question was structured within a PICO (Problem/Population, Intervention, Comparison and Outcome) framework. Population of interest included patients affected by histologically proven esophageal adenocarcinoma/squamous cells cancer. The intervention was robotic transthoracic esophagectomy, and the comparator was open esophagectomy and laparoscopic esophagectomy, respectively. 

Outcome measures were divided in short- and long-term outcomes. Short-term outcomes encompassed postoperative complications rate, in terms of anastomotic leakage, postoperative bleeding, wound infection, pneumonia, recurrent laryngeal nerves (RLN) paralysis, chylotorax, reoperation rate and overall mortality, intraoperative outcomes (mean blood loss, operative time and conversion), oncologic outcomes (harvested nodes, R0 resection rate) and recovery outcomes (length of hospital stay). Long-term outcomes included recurrences and 5-year overall survival.

The literature search and study selection were performed independently by two reviewers. In case of disagreement, a third investigator was consulted and an agreement was reached by consensus.

### 2.2. Data Extraction and Risk of Bias Assessment

The following data were extracted from each study: first author, year of publication, study design, sample size, demographic characteristics, number of patients in each surgical group, gender, mean age, mean BMI (Body Mass Index), ASA (American Society of Anesthesiologists) Score, tumor stage according to UICC (Union for International Cancer Control), preoperative radio-chemotherapy rate, mean blood loss, operative time, conversion, anastomotic leakage, postoperative bleeding, wound infection, pneumonia, recurrent laryngeal nerves (RLN) paralysis, chylotorax, reoperation rate and overall mortality, harvested nodes, R0 resection rate, length of hospital stay, recurrence rate and 5-years overall survival. In order to assess overall mortality, we considered in-hospital mortality and 30-days and 90-days mortality, performing a sum of these data in each group. 

Study quality assessment of the included studies was performed with the Newcastle Ottawa Scale (NOS) [20]. This scoring system encompasses three major domains (selection, comparability and exposure), with scores between 0 (lowest quality) to 9 (highest quality). In case of Randomized Controlled Trial (RCTs), the risk of bias was evaluated according to the Cochrane Collaboration Tool for assessing risk of bias [21]. According to this scoring system, seven domains were evaluated as “Low risk of bias” or “High risk of bias” or “Unclear” according to reporting on sequence generation, allocation concealment, blinding of participants, blinding of outcome assessment, incomplete outcome data, selective outcome reporting, and other potential threats to validity.

### 2.3. Statistical Analysis

Statistical analysis was performed using Comprehensive Meta-Analysis (Version 2.2, Biostat Inc, Englewood, NJ, USA, 2005). In order to provide a complete update on robotic surgery for esophageal cancer, two different group analyses were performed: robotic vs. laparoscopic and robotic vs. open approach.

Furthermore, for each meta-analysis, two subgroup analyses were performed dividing the studies according to the surgical procedure (Ivor-Lewis esophagectomy or McKeown esophagectomy). Finally, a sensitivity analysis excluded studies applying a hybrid approach (robotic abdominal phase and laparoscopic/open thoracic phase) and studies which did not specify the surgical procedure.

The odds ratio (OR) along with 95% confidence interval was used as effect estimate for dichotomous outcomes. In case of rare events, the risk difference (RD) with corresponding 95%CI were calculated, maintaining analytic consistency and including all available data, in accordance with Messori et al. [22]. In case studies reporting median, range and sample size, or studies reporting median and quartile ranges, the means and standard deviations were estimated according to Shi, Luo and Wan [23,24,25]. In studies reporting mean values without standard deviation, the latter was imputed, according to Furukawa et al. [26]. The overall effect was tested using Z scores and significance was set at *p* < 0.05. The summary estimate was computed under a random effects assumption as per DerSimonian and Laird [27]. A conservative random effect model was chosen a priori in consideration of foreseen heterogeneity among the included studies. The heterogeneity among the studies was quantified by the I^2^ statistic, with I^2^ values < 25%, between 25–50%, and >50% indicating respectively low, moderate, and high heterogeneity [28,29]. The presence of publication bias was investigated through a funnel plot where the summary estimate of each study (OR) was plotted against the standard error as a measure of study precision. In addition to visual inspection, funnel plot symmetry was tested using the Egger’s linear regression method [30]. *p* values ≤ 0.05 were considered statistically significant.

## 3. Results

### Study Selection

**Figure 1:**
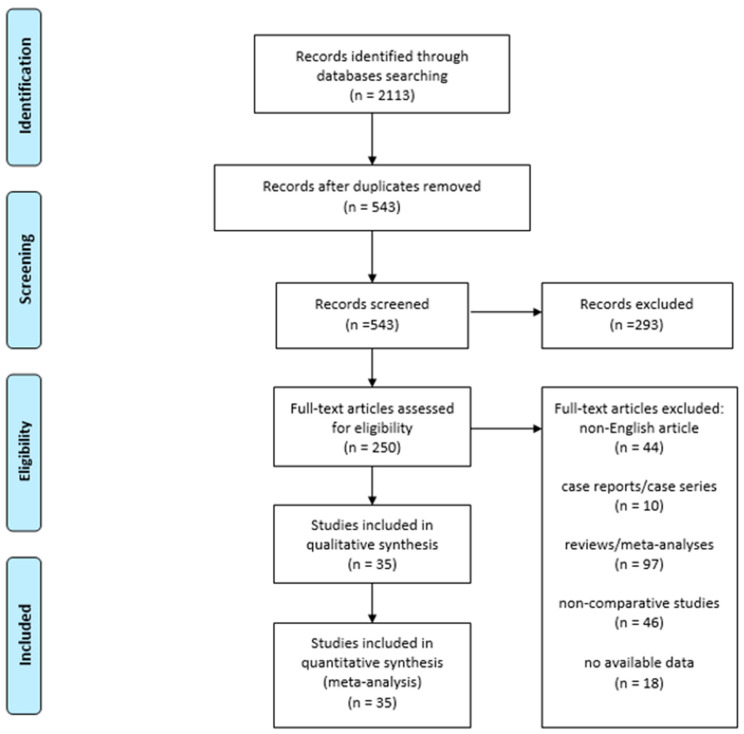
PRISMA Flowchart.

The electronic search returned a total of 2113 results. After duplicates removal, 543 studies entered first-level screening. A total of 507 studies were excluded for the following reasons: 44 were written in a language other than English, 10 were case reports/case series, 97 were reviews, 46 were non-comparative studies, 293 were off-topic and 18 did not provide any usable data. Thus, 35 studies were included in the final analysis, out of which 20 compared robotic vs. laparoscopic surgery, 11 compared robotic vs. open esophagectomy and 4 reported on a three-arms comparison (robotic vs. laparoscopic vs. open) [20,21,22,23,24,25,26,27,28,29,30,31,32,33,34,35,36,37,38,39,40,41,42,43,44,45,46,47,48,49,50,51,52,53,54]. From the latter [54], it was possible to extract only data about the comparison between robotic and laparoscopic esophagectomy. Record selection is illustrated in the PRISMA flowchart (Figure 1). Inter-rater agreement was perfect (κ = 1).

## 4. Robotic Versus Laparoscopic Esophagectomy

### 4.1. Study Characteristics

All were prospective (*n* = 5) or retrospective studies (*n* = 18) [20,21,22,23,29,32,33,37,40,42,43,44,45,46,48,51,53], reporting on 11,779 patients, of whom 3832 underwent robotic esophagectomy and 7947 laparoscopic esophagectomy. The characteristics of the included studies are summarized in Table 1. 

Ivor-Lewis procedure was performed in four studies [39,49,51,56], McKeown esophagectomy in ten studies [34,42,43,53,54,57,58,59,60,61] while nine studies did not specify the intervention [31,32,33,40,47,48,50,52,55].

### 4.2. Risk of Bias Assessment

All studies had NOS quality scores greater than 6, indicating fair methodological quality. Specifically, thirteen studies had NOS quality score = 7; ten studies had NOS quality score = 8. The NOS quality score is represented in Supplementary Table S1. No RCTs comparing robotic and laparoscopic transthoracic esophagectomy were published.

### 4.3. Short Term-Outcomes

**Figure 2:**
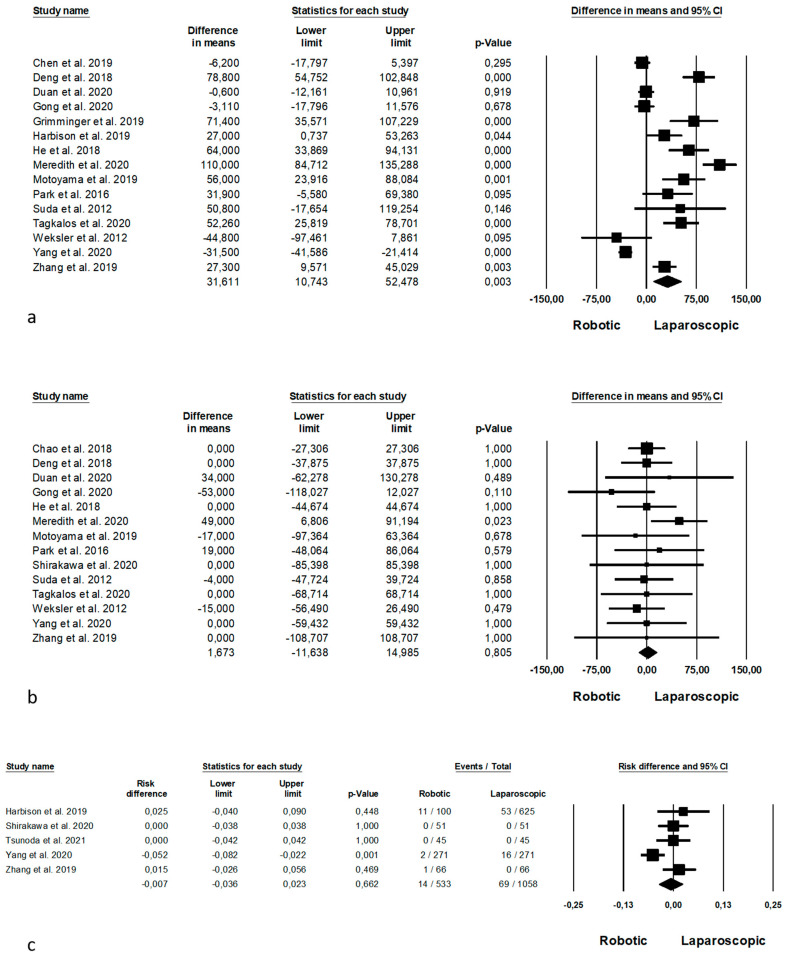
Robotic versus laparoscopic surgery: intraoperative outcomes. (**a**) operative time; (**b**) estimated blood loss; (**c**) conversion.

Intraoperative outcomes are shown in Figure 2. Operative time was reported by 15 Authors [23,25,26,28,29,30,31,35,37,40,45,46,49,54,57] on 2690 procedures (which of 1089 robotic and 1601 laparoscopic), demonstrating a lower operative time in the laparoscopic group (MD = 31, *p* = 0.003, 95%CI 10.743; 52.478), with a significant heterogeneity among the studies (I^2^ = 93.720%, *p* < 0.0001). Estimated blood loss was analysed by 14 Authors [21,23,28,29,33,37,38,39,42,48,50,51,53,57], on 1977 procedures (which of 995 robotic and 982 laparoscopic), demonstrating no significant differences between the two approaches (MD = 1.673, *p* = 0.805, 95%CI −11.638; 14.985), with no heterogeneity among the studies (I^2^ = 0%, *p* = 0.760). Number of conversion was reported by 5 Authors [33,47,50,56,57] on 1591 procedures (which of 533 robotic and 1058 laparoscopic), with no significant difference between the two groups (RD = −0.007, *p* = 0.662, 95%CI −0.036; 0.023), but with a significant heterogeneity among the studies (I^2^ = 61.532%, *p* = 0.034).

**Figure 3:**
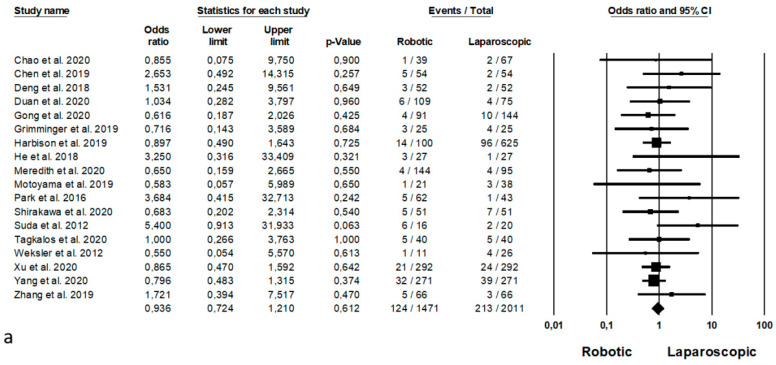

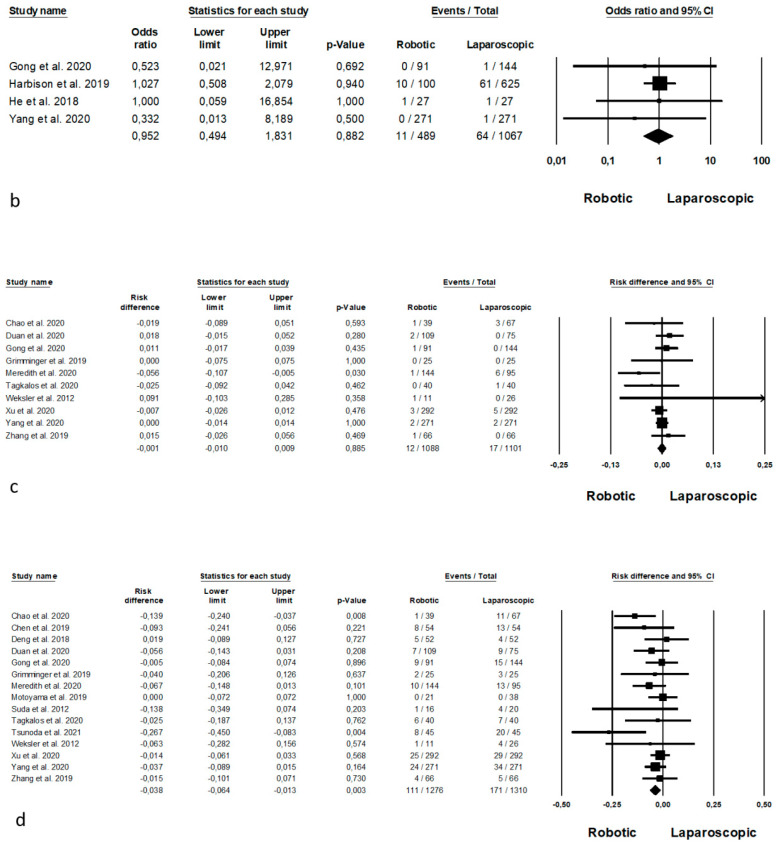

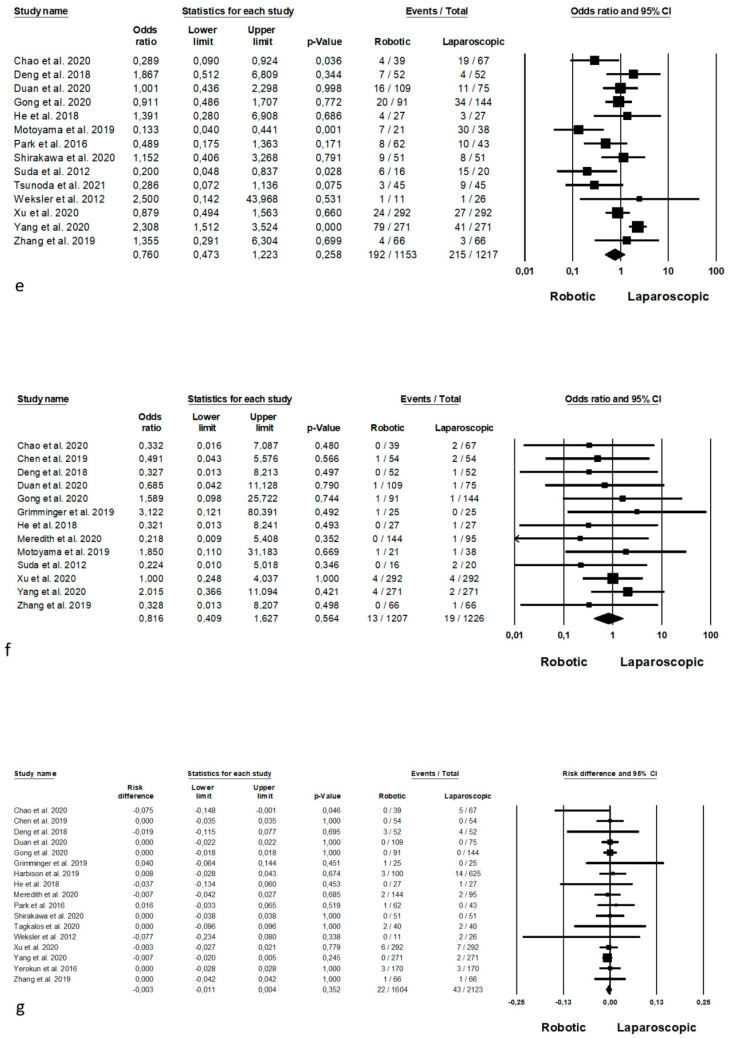
Robotic versus laparoscopic surgery: postoperative complications. (**a**) anastomotic leakage; (**b**) postoperative bleeding; (**c**) wound infection; (**d**) pneumonia; (**e**) RLN paralysis; (**f**) chylothorax; (**g**) mortality.

Statistical analysis for postoperative complications are shown in Figure 3. Anastomotic leakage was analysed by 18 Authors [22,23,28,29,32,33,37,38,39,42,43,45,48,50,51,53,54,57] on 3482 procedures (1471 robotic and 2011 laparoscopic), with no statistical differences between the two approaches (OR = 0.936, *p* = 0.612, 95%CI 0.724, 1.210) and no significant heterogeneity among the studies (I^2^ = 0%, *p* = 0.871). Postoperative bleeding was reported by 4 Authors [33,34,57,61] on 1556 procedures (489 robotic and 1067 laparoscopic), demonstrating no significant differences between the two groups (OR = 0.952, *p* = 0.882, 95%CI 0.494, 1.831) and no significant heterogeneity among the studies (I^2^ = 0%, *p* = 0.898). Postoperative wound infection was analysed by 10 Authors [32,38,49,51,53,54,56,57,60,61] on 2189 procedures (1088 robotic and 1101 laparoscopic), with no significant differences between the two approaches (RD = −0.001, *p* = 0.885, 95%CI −0.010; 0.009) and no significant heterogeneity among the studies (I^2^ = 7.881%, *p* = 0.370). Pneumonia was reported by 15 Authors [27,29,32,38,39,40,42,43,45,48,50,51,53,54,57] on 2586 procedures (1276 robotic and 1310 laparoscopic), with a lower number of pneumonias in the robotic group (RD = −0.038, *p* = 0.003, 95%CI −0.064; −0.013) and no significant heterogeneity among the studies (I^2^ = 0%, *p* = 0.726). 

RLN paralysis was reported by 14 Authors [23,29,32,33,37,38,40,42,45,48,50,51,53,57] on 2370 procedures (1153 robotic and 1217 laparoscopic), with no significant differences between the two approaches (OR = 0.760, *p* = 0.258, 95%CI 0.473, 1.223), but with a significant heterogeneity among the studies (I^2^ = 69.109%, *p* < 0.0001). Chylothorax was analysed by 13 Authors [23,27,29,32,38,43,45,48,50,51,53,54,57] on 2433 procedures (1207 robotic and 1226 laparoscopic), with no significant differences between the two groups (OR = 0.816, *p* = 0.564, 95%CI 0.409, 1.627), and no significant heterogeneity among the studies (I^2^ = 0%, *p* = 0.954). Mortality was analysed by 17 Authors [22,23,28,31,32,36,38,41,43,44,45,46,48,50,51,53,54] including 3727 patients (1604 robotic and 2123 laparoscopic) with no differences between the two groups (RD = −0.003, *p* = 0.352, 95%CI −0.011; 0.004) and no significant heterogeneity among the studies (I^2^ = 0%, *p* = 0.962). It was not possible to assess the reoperation rate because no studies reported this data.

**Figure 4:**
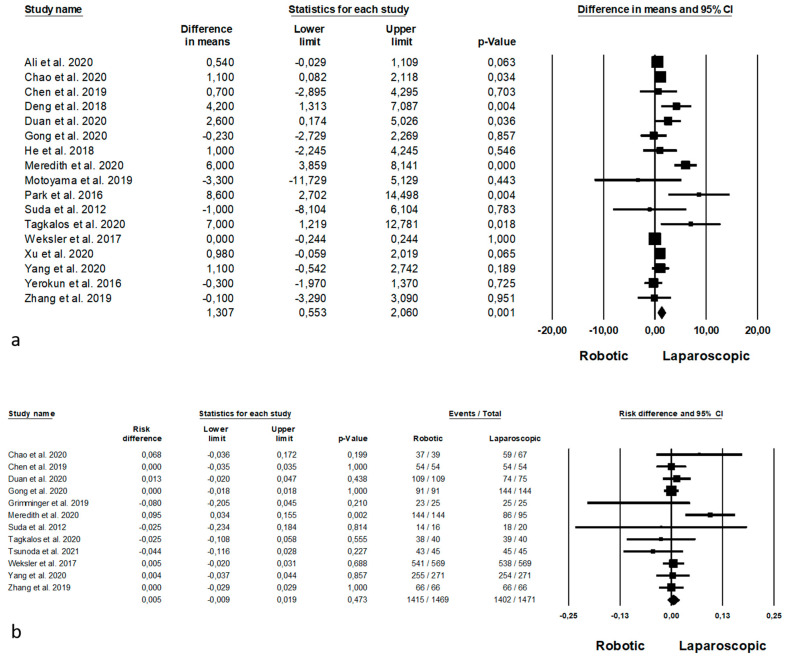
Robotic versus laparoscopic surgery: oncologic outcomes. (**a**) Number of harvested nodes; (**b**) R0 resection.

Oncologic outcomes are shown in Figure 4. Mean number of harvested nodes was reported by 17 Authors [31,34,38,40,43,48,49,52,53,54,55,56,57,58,59,60,61] on 10,707 procedures (which of 3566 robotic and 7141 laparoscopic), demonstrating a higher number in the harvested nodes during the robotic approach (MD = 1.307, *p* = 0.001, 95%CI 0.553; 2.060), with a significant heterogeneity among the studies (I^2^ = 74.857%, *p* < 0.0001). The number of complete resection (R0 resection) was reported by 12 Authors [32,38,48,49,50,52,53,56,57,58,60,61] on 2940 procedures (which of 1469 robotic and 1471 laparoscopic), with no significant differences between the two procedures (RD = 0.005, *p* = 0.473, 95%CI −0.009; 0.019), and no significant heterogeneity among the studies (I^2^ = 20.790%, *p* = 0.258).

**Figure 5:**
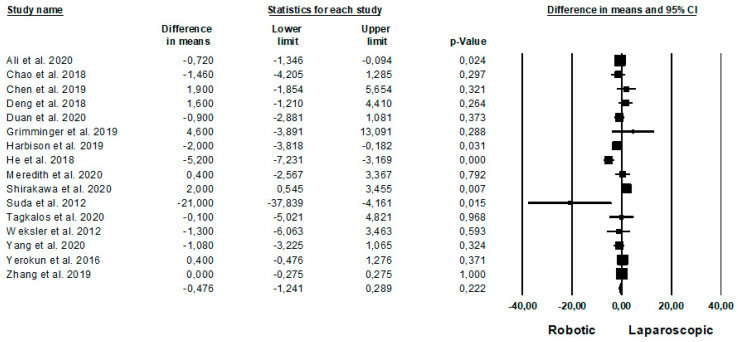
Robotic versus laparoscopic surgery: length of hospital stay.

Length of hospital stay was represented in Figure 5. This data was reported by 16 Authors [20,21,22,23,28,36,37,38,41,43,45,46,48,50,51,54], on 9642 patients (2713 robotic and 6749 laparoscopic), demonstrating no differences between the two approaches (MD = −0.476, *p* = 0.289, 95%CI −1.241; 0.289), with a significant heterogeneity among the studies (I^2^ = 72.303%, *p* < 0.0001).

## 5. Long-Term Outcomes

**Figure 6:**
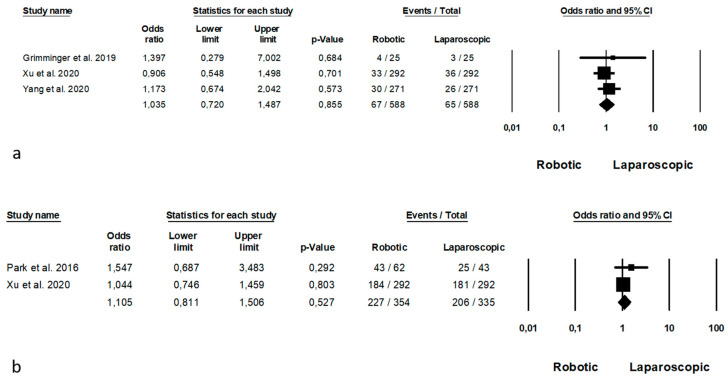
Robotic versus laparoscopic surgery: long-term outcomes. (**a**) recurrences; (**b**) 5-years overall survival.

Long-term outcomes are summarized in Figure 6. Recurrences were analysed by 3 Authors [32,54,57] on 1176 patients (588 in each arm), with no significant differences between the two groups (OR = 1.035, *p* = 0.855, 95%CI 0.720, 1.487) and no significant heterogeneity among the studies (I^2^ = 0%, *p* = 0.742). The 5-years overall survival was reported by 2 Authors [43,54], with no significant differences between the two groups (OR = 1.105, *p* = 0.527, 95%CI 0.811, 1.506) and no significant differences among the studies (I^2^ = 0%, *p* = 0.380).

## 6. Subgroup Analysis

### 6.1. Fully Robotic vs. Fully Laparoscopic Procedures

Excluding the two studies in which the surgical procedures was not clearly described [52] or in which a hybrid approach was adopted [33,55], this subgroup analysis included 21 studies [31,32,33,34,38,40,42,43,47,48,49,50,51,53,54,56,57,58,59,60,61].

It was not possible to obtain data about blood loss, wound infection, postoperative pneumonia, RLN paralysis and chylothorax because the above-mentioned study did not report these data.

Of the remaining outcomes, the subgroup analysis confirmed the results of the main analysis in terms of operative time (lower in the laparoscopic group, MD = 32, *p* = 0.004, 95%CI 9.983; 53.978), conversion (RD = −0.011, *p* = 0.495, 95%CI −0.043; 0.021), anastomotic leakage (OR = 0.945, *p* = 0.693, 95%CI 0.711; 1.254), bleeding (OR = 0.587, *p* = 0.555, 95%CI 0.100; 3.443), mortality (RD = −0.004, *p* = 0.283, 95%CI −0.012; 0.003), harvested nodes (MD = 1.748, *p* < 0.0001, 95%CI 0.795; 2.701), R0 resection (RD = 0.005, *p* = 0.528, 95%CI −0.011; 0.022) and hospital stay (MD = −0.462, *p* = 0.318, 95%CI −1.369; 0.444).

### 6.2. McKeown Esophagectomy

After excluding four studies about Ivor-Lewis procedure [38,49,51,56] and other nine in which Ivor-Lewis and Mckeown were not separately analysed [31,32,33,40,47,48,50,52,55], ten studies [34,42,43,53,54,57,58,59,60,61] were included in the subgroup analysis according to Mckeown procedure.

Of intraoperative data, no difference was found between robotic and laparoscopic approach in terms of estimated blood loss (MD = −1.370, *p* = 0.876, 95%CI −18.547; 15.808, respectively). Interestingly, in this subgroup analysis there was no difference in term of operative time (MD = 11.262, *p* = 0.334, 95%CI −11.595; 34.118), conversely to the main analysis. It was not possible to extract data about conversion because only one study was about McKeown esophagectomy.

Of postoperative complications, the subgroups analysis confirmed no significant differences were between the two approaches in terms of anastomotic leakage (OR = 0.928, *p* = 0.659, 95%CI 0.667, 1.291), bleeding (OR = 0.587, *p* = 0.555, 95%CI 0.100; 3.443), wound infection (RD = −0.001, *p* =0.878, 95%CI −0.009; 0.011), RLN paralysis (OR = 0.994, *p* = 0.981, 95%CI 0.609, 1.623), chylothorax (OR = 0.880, *p* = 0.753, 95%CI 0.397; 1.949) and mortality (RD = −0.004, *p* = 0.285, 95%CI −0.013; 0.004). Similarly, a significant difference was confirmed in terms of postoperative pneumonia between the two groups in favour of robotic surgery (RD = −0.035, *p* = 0.028, 95%CI −0.066; −0.004).

Confirming the data of the main analysis, robotic surgery was associated with a higher number of harvested nodes (MD = 1.445, *p* = 0.001, 95%CI 0.572; 2.318), while no differences were found in terms of R0 resection and recurrences (RD = 0.004, *p* = 0.593, 95%CI −0.010; 0.017 and OR = 1.018, *p* = 0.925, 95%CI 0.701; 1.478, respectively).

Finally, no significant differences were found in terms of length of hospital stay between the two approaches (MD = −1.058, *p* = 0.316, 95%CI −3.125; 1.009).

### 6.3. Ivor-Lewis Esophagectomy

The subgroup analysis on Ivor-Lewis esophagectomy included four studies [38,49,51,56].

The sub-analysis of intraoperative data confirmed that there was no difference between the two approaches in terms of estimated blood loss (MD = 11.916, *p* = 0.513, 95%CI −23.794; 47.626, respectively). On the contrary, subgroup analysis showed no difference in terms of operative time (MD = 39.990, *p* = 0.112, 95%CI −9.367; 89.347) between the two approaches. It was not possible to extract data about conversions because only one study was about the Ivor-Lewis procedure.

Of postoperative complications, no significant differences were found in terms of anastomotic leakage (OR = 0.956, *p* = 0.907, 95%CI 0.446; 2.049), wound infection (RD = −0.014, *p* = 0.531, 95%CI −0.059; 0.030), RLN paralysis (OR = 1.553, *p* = 0.524, 95%CI 0.401; 6.022), chylothorax (OR = 0.267, *p* = 0.255, 95%CI 0.028; 2.597) and mortality (RD = −0.006, *p* = 0.652, 95%CI −0.031; 0.019), confirming the data of the main analysis. Interestingly, rate of postoperative pneumonia (RD = −0.042, *p* = 0.123, 95%CI −0.096; 0.011) did not differ between the two approaches. No data were extracted about postoperative bleeding because no studies about Ivor-Lewis esophagectomy reported this data.

About oncologic outcomes, no difference was found in terms of R0 resection (RD = 0.024, *p* = 0.473, 95%CI −0.042; 0.091) and differently to the main analysis, no difference was found on number of harvested nodes (MD = 4.091, *p* = 0.077, 95%CI −0.450; 8.631). No data were extracted about recurrence because of the absence of studies about Ivor-Lewis esophagectomy analysing this aspect.

No differences in terms of length of hospital stay was found between the two approaches (MD = −0.001, *p* = 0.993, 95%CI −0.274; 0.272).

### 6.4. Publication Bias

Forest plots were symmetrical across outcomes and the Egger’s test was not suggestive of publication bias, except for the mean number of harvested nodes and operative time, in which visual inspection suggested an asymmetric distribution of studies around the mean and the Egger’s test confirmed significant publication bias (*p* = 0.01 and *p* = 0.006, respectively). Funnel plots are provided in Supplementary Figures S1–S4.

## 7. Robotic Versus Open Esophagectomy

### 7.1. Study Characteristics

Seven retrospective [35,41,44,45,52,61,62] and four prospective cohort studies [37,39,46,63], and two RCTs were identified [11,64], reporting on 4485 patients, out of whom 1919 underwent robotic esophagectomy and 2566 open esophagectomy. The characteristics of the included studies are detailed in Table 2.

About surgical intervention, all the surgical interventions of the included studies were performed with a fully robotic approach, except for the study by Rolff et al. [45], in which an hybrid procedure (robotic approach to the abdomen and open approach to the thorax) was used. Two articles reported on Ivor-Lewis procedure [37,39], one on McKeown esophagectomy [61] while ten did not provide relevant data to allow subgroup analysis [11,35,41,44,45,46,52,62,63,64].

### 7.2. Risk of Bias Assessment

All studies had NOS quality scores greater than 6, indicating that all these studies had fair methodological quality. Specifically, seven studies had NOS quality score = 8; six had NOS quality score = 7. The NOS quality score is represented in Table 2. The two included RCTs [11,64] had low risk of bias.

### 7.3. Short-Term Outcomes

**Figure 7:**
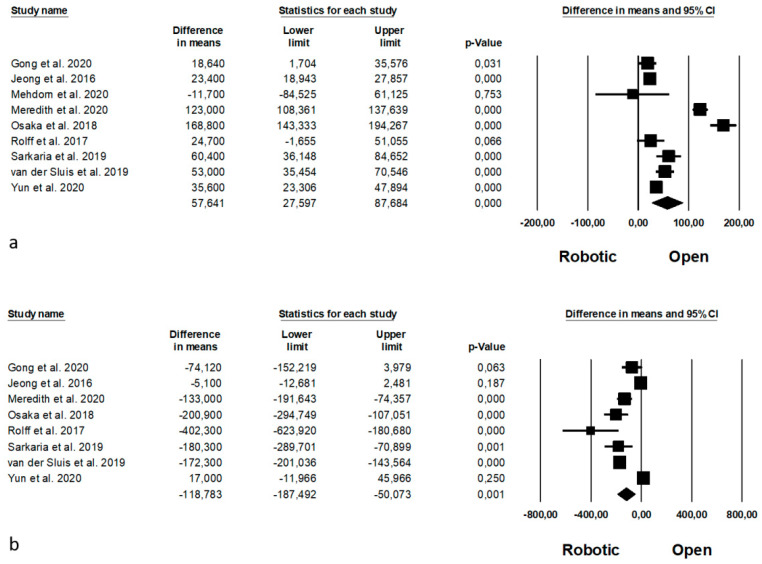
Robotic versus open surgery: intraoperative outcomes. (**a**) operative time; (**b**) estimated blood loss.

Intraoperative outcomes are shown in Figure 7. Operative time was reported by 9 Authors [11,35,37,38,41,45,46,61,63] on 1982 procedure (which of 668 robotic and 1314 open), demonstrating a lower operative time in the open group (MD = 57, *p* < 0.0001, 95%CI 27.597; 87.684), with a significant heterogeneity among the studies (I^2^ = 97.190%, *p* < 0.0001). Estimated blood loss was analysed by 8 Authors [11,35,39,41,45,46,61,63], on 1960 procedures (which of 657 robotic and 1303 open), demonstrating a significantly lower blood loss in the robotic group (MD = −118.783, *p* = 0.001, 95%CI −187.492; −50.073), with a significant heterogeneity among the studies (I^2^ = 96.086%, *p* < 0.0001).

**Figure 8:**
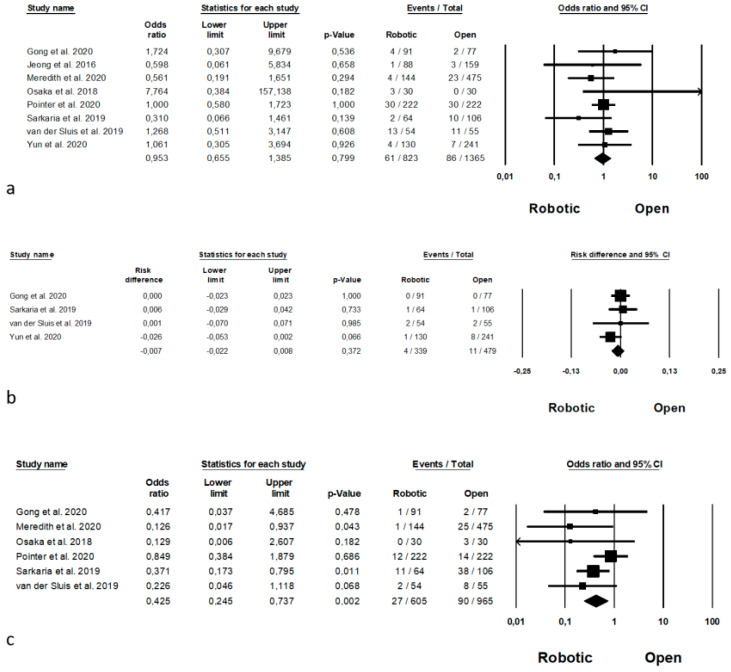

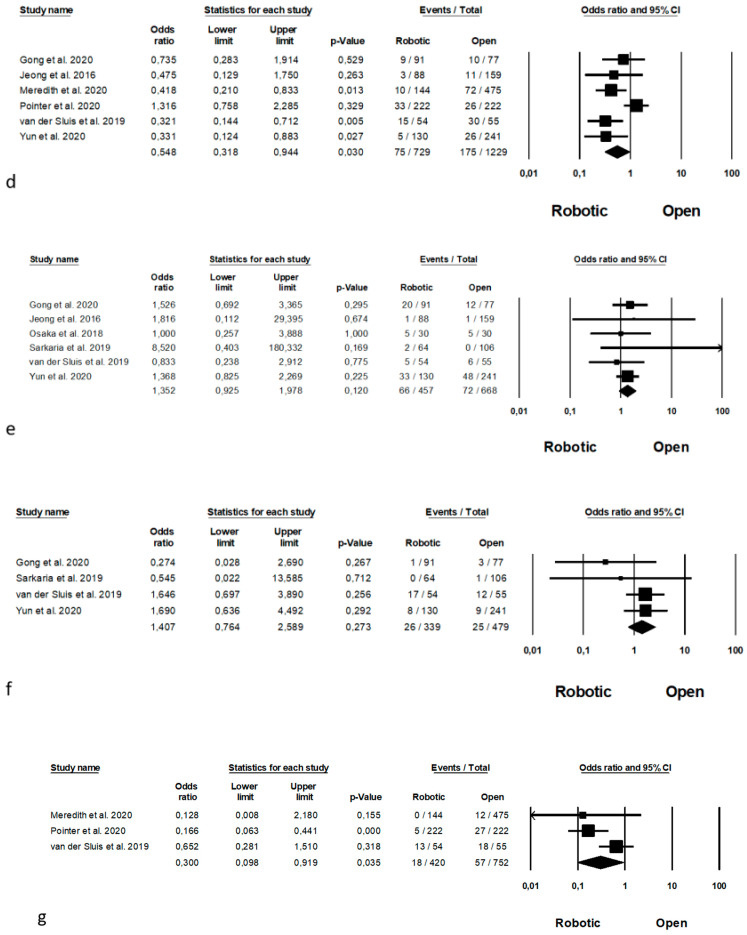

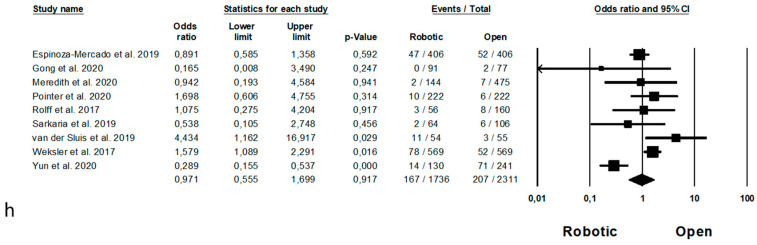
Robotic versus open surgery: postoperative complications. (**a**) anastomotic leakage; (**b**) postoperative bleeding; (**c**) wound infection; (**d**) pneumonia; (**e**) RLN paralysis; (**f**) chylothorax; (**g**) re-operation rate; (**h**) mortality.

Postoperative complications are shown in Figure 8. Anastomotic leakage was analysed by 8 Authors [11,35,39,41,44,46,61,63] on 2188 procedures (823 robotic and 1365 open), with no statistical differences between the two approaches (OR = 0.953, *p* = 0.799, 95%CI 0.655; 1.385) and no significant heterogeneity among the studies (I^2^ = 0%, *p* = 0.556). Postoperative bleeding was reported by 4 Authors [11,46,61,63] on 818 procedures (339 robotic and 479 open), demonstrating no significant differences between the two groups (RD = −0.007, *p* = 0.372, 95%CI −0.022; 0.008) and no significant heterogeneity among the studies (I^2^ = 0%, *p* = 0.439). Postoperative wound infection was analysed by 6 Authors [11,39,41,44,46,61] on 1570 procedures (605 robotic and 965 open), with a significant differences between the two approaches in favour of robotic surgery (OR = 0.425, *p* = 0.002, 95%CI 0.245; 0.737) and no significant heterogeneity among the studies (I^2^ = 11.051%, *p* = 0.345). Pneumonia was reported by 6 Authors [11,35,39,44,61,63] on 1958 procedures (729 robotic and 1229 open), with a lower number of pneumonias in the robotic group (OR = 0.548, *p* = 0.03, 95%CI 0.318; 0.944), but with a significant heterogeneity among the studies (I^2^ = 61.247%, *p* = 0.024). Pneumonia rate are expressed in percentage and are available in Supplementary Table S3.

RLN paralysis was reported by 6 Authors [11,35,41,46,61,63] on 1125 procedures (457 robotic and 668 open), with no significant differences between the two approaches (OR = 1.352, *p* = 0.120, 95%CI 0.925, 1.978) and no significant heterogeneity among the studies (I^2^ = 0%, *p* = 0.807). Chylothorax was analysed by 4 Authors [11,46,61,63] on 818 procedures (339 robotic and 479 open), with no significant differences between the two groups (OR = 1.407, *p* = 0.273, 95%CI 0.764; 2.589), and no significant heterogeneity among the studies (I^2^ = 0%, *p* = 0.463). Re-operations were reported by 3 Authors [11,38,44] on 1172 procedures (420 robotic and 752 open), with a significant differences in favour of robotic surgery approaches (OR = 0.300, *p* = 0.035, 95%CI 0.098, 0.919) with no significant heterogeneity among the studies (I^2^ = 58.531%, *p* = 0.09). Mortality was analysed by 9 Authors [11,39,44,45,46,52,61,62,63] including 4047 patients (1736 robotic and 2311 open) with no differences between the two groups (OR = 0.971, *p* = 0.917, 95%CI 0.555; 1.699) and no significant heterogeneity among the studies (I^2^ = 72.556%, *p* < 0.0001).

**Figure 9:**
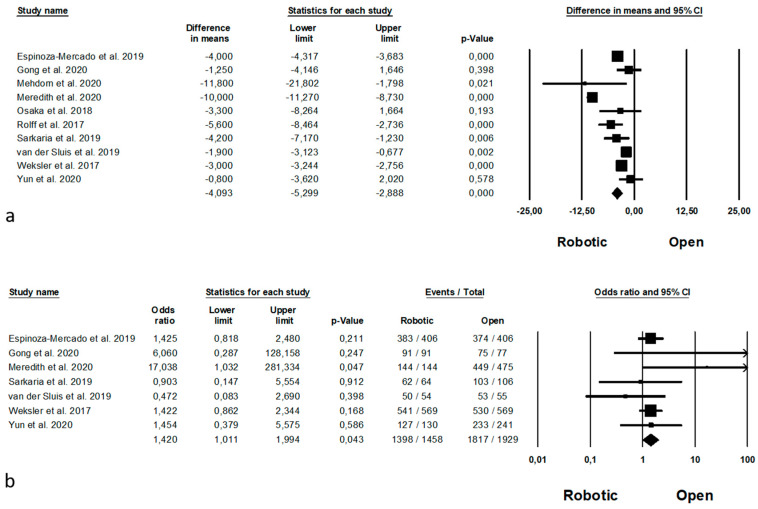
Robotic versus open surgery: oncologic outcomes. (**a**) number of harvested nodes; (**b**) R0 resection.

Oncologic outcomes are shown in Figure 9. Mean number of harvested nodes was reported by 10 Authors [11,37,38,41,45,46,52,61,62,63] on 3685 procedures (which of 1555 robotic and 2130 open), demonstrating a higher number of the harvested nodes during the robotic approach (MD = −4, *p* < 0.0001, 95%CI −5.299; −2.888), with a significant heterogeneity among the studies (I^2^ = 94.059%, *p* < 0.0001). The number of complete resection (R0 resection) was reported by 7 Authors [11,39,46,52,61,62,63] on 3387 procedures (which of 1458 robotic and 1929 open), with a significantly higher number of R0 resection in the robotic group (OR = 1.420, *p* = 0.043, 95%CI 1.011; 1.994), and no significant heterogeneity among the studies (I^2^ = 0%, *p* = 0.462). Oncologic outcomes are expressed as means and standard deviation (harvested nodes) and percentage (R0 resection rate) in Supplementary Tables S4 and S5, respectively.

**Figure 10:**
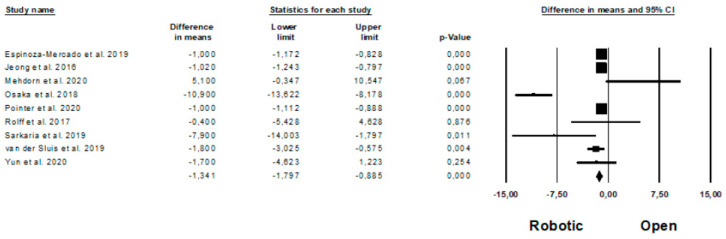
Robotic versus open surgery: length of hospital stay.

Length of hospital stay was represented in Figure 10. This data was reported by 9 Authors [11,35,37,41,44,45,46,62,63], on 2549 patients (1110 robotic and 1439 open), demonstrating a shorter length of hospital stay in the robotic group (MD = −1.341, *p* < 0.0001, 95%CI −1.797; −0.885), with a significant heterogeneity among the studies (I^2^ = 87.169%, *p* < 0.0001).

## 8. Long-Term Outcomes

**Figure 11:**
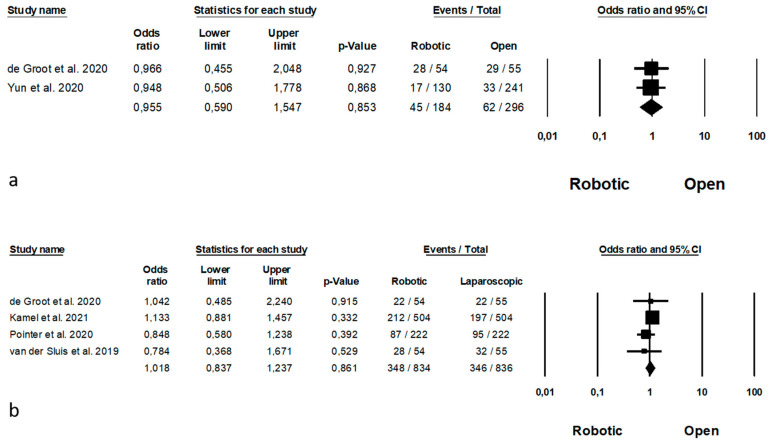
Robotic versus open surgery: long-term outcomes. (**a**) recurrences; (**b**) 5-years overall survival.

Long-term outcomes are represented in Figure 11. Recurrences was analysed by 2 Authors [63,64] on 480 patients (184 robotic and 296 open), with no significant differences between the two groups (OR = 0.955, *p* = 0.853, 95%CI 0.590; 1.547) and no significant heterogeneity among the studies (I^2^ = 0%, *p* = 0.971). The 5-years overall survival was reported by 4 Authors [11,36,44,64] on 1670 procedures (834 robotic and 836 open), with no significant differences (OR = 1.018, *p* = 0.861, 95%CI 0.837; 1.237) and no significant heterogeneity among the studies (I^2^ = 0%, *p* = 0.562).

## 9. Subgroup Analysis

### 9.1. Fully Robotic vs. Open Procedures

To perform this subgroup analysis only the study by Rolff et al. [45] and Weksler et al. [52] were excluded. Thus, the subgroup analysis included eleven studies [11,35,37,38,41,44,46,61,62,63,64].

About intraoperative outcomes, subgroup analysis confirmed a significantly lower operative time (MD = 61.676, *p* < 0.0001, 95%CI 28.905; 94.448) in the open surgery group and lower estimated blood estimated blood loss (MD = −100.742, *p* = 0.004, 95%CI −169.793; −31.692) in the robotic group.

About postoperative complications, only data about mortality could be extracted, without a significant difference between the two approaches (OR = 0.855, *p* = 0.668, 95%CI 0.418; 1.748).

Only data regarding harvested nodes could be extracted in terms of oncologic outcomes in the subgroup analysis, confirming a significant difference between the two approaches in favour of robotic approach (MD = 3.783, *p* = 0.002, 95%CI 1.385; 6.180).

Hospital stay was confirmed to be shorter in the robotic group (MD = −1.353, *p* < 0.0001, 95%CI −1.814; −0.892).

### 9.2. McKeown Esophagectomy

It was not possible to perform a subgroup analysis because only one study [61] reported data about the comparison between robotic and open McKeown esophagectomy.

### 9.3. Ivor-Lewis Esophagectomy

Ivor-Lewis esophagectomy was described by only two studies [37,38]. It was possible to perform a subgroup analysis about operative time and harvested nodes. Analysis of operative time showed no significant differences between the two approaches (MD = 60.568, *p* = 0.367, 95%CI −71.084; 192.219). On the contrary, the analysis on harvested nodes confirmed the higher number of this parameter in the robotic group (MD = 10.029, *p* < 0.0001, 95%CI 8.768; 11.289).

### 9.4. Publication Bias

Plot analysis showed a symmetrical distribution of the studies evaluating all the analysed outcomes, without evidence of publication bias by the Egger’s test. Funnel plots are shown in Supplementary Figures S5–S8.

## 10. Discussion

The standard treatment of the esophageal cancer is nowadays considered radical esophagectomy with a complete lymphadenectomy whenever this is feasible [65]. Minimally invasive approaches have emerged over the last decades, with the objective to minimize surgical trauma and optimize postoperative outcomes [65].

Minimally invasive esophagectomy (MIE) has gained momentum because of evidence suggesting lower postoperative complication rate and similar oncologic results compared to conventional thoracotomy approaches [66,67,68].

More recently, Robot-Assisted Minimally Invasive Esophagectomy (RAMIE) was introduced as an alternative minimally invasive method which may allow improved view of thoracic structures and increased precision [69]. Nevertheless, the presumed advantages of the robotic surgery are still under debate [69,70,71]. In this setting, three meta-analysis tried to assess if the robotic approach could be considered the best treatment to the esophageal cancer [70,71,72]. In a network meta-analysis on 98 studies and 32,315 patients, Siaw-Acheampong et al. [70] compared all combinations of open, laparoscopic and robotic approaches to transthoracic esophagectomy. Their results demonstrated that compared with open surgery, both laparoscopic and robotic approaches were associated with less blood loss, significantly lower rates of pulmonary complications, shorter hospital stay and higher mean of harvested nodes, concluding that minimally invasive approaches were related with better postoperative outcomes with no compromise in oncologic results. Regarding the comparison between laparoscopic and robot-assisted approach, Zheng et al. [71] identified fourteen studies with a total of 2887 patients included in the final an analysis. The Authors demonstrated that RAMIE was associated with a lower incidence of pneumonia and vocal cord palsy than MIE, but still be associated with longer operative time. Additionally, Li et al. [72] demonstrated in a meta-analytic comparison between 866 patients in the RAMIE group and 883 patients in the MIE group that RAMIE yielded significantly higher number of lymph nodes. Both Authors independently concluded that RAMIE could be a standard treatment for transthoracic approach to esophageal cancer. From that knowledge, in the last two years fifteen new studies have been published comparing robotic approach with the other surgical techniques, confirming the fervid interest in this topic.

By pooling respectively 11,779 comparing robotic versus laparoscopic and 4485 robotic versus open esophagectomy we are able to provide pros and cons of the robotic approach.

Robotic approach appears to provide some advantages over open approach. In fact, our results showed that robotic approach is clearly superior over open surgery in terms of intraoperative outcomes (less blood loss *p* = 0.001), postoperative complications (lower wound infection rate, *p* = 0.002; pneumonia rate, *p* = 0.03; re-operation rate *p* = 0.03) and oncologic outcomes (mean number of harvested nodes, *p* < 0.0001; R0 resection rate, *p* = 0.043). The possible explanation of these better oncologic results could lay in the magnification of the images and in the finer dissection movements properly related to the robotic technology. Considering the current literature, these results are completely in accordance with the previous network meta-analysis by Siaw-Acheampong et al. [70], confirming the advantages of the robotic approach over open technique. On the contrary, no disadvantages were associated with the robotic surgery, except for operative time (longer in the robotic group, *p* < 0.0001), but with no association with non-surgical postoperative complications. Finally, we can assess the safety of robotic approach, guaranteed by the absence of significant differences over open surgery in terms of postoperative complications. Additional conclusion could be provided by the comparison between robotic and conventional laparoscopic approach. Robotic approach seemed to be superior to conventional laparoscopy in terms of oncologic outcomes (mean number of harvested nodes obtained, *p* = 0.001) and postoperative complications (incidence of pneumonia after surgery, *p* = 0.003). Even in this case robotic surgery has the only disadvantage of operative time (shorter in the laparoscopic group, *p* = 0.003), but this data was not associated with increased postoperative morbidities.Our results are in accordance with the results of the meta-analysis by Zheng et al. [71] in terms of longer operative time in the robotic group. Similarly pneumonia rate was lower in the robotic group, and this data has been confirmed by our analysis. Comparing our results with the results obtained by the meta-analysis by Li et al. [72], it is easy to notice an accordance in the setting of number of yielded lymph nodes, significantly higher in the robotic group. On the contrary, Li et al. [72] demonstrated a lower blood loss in the robotic group, in our meta-analysis there was no significant differences between the two groups.

Finally, it is important to highlight that our results were confirmed by the subgroups analyses both for robotic versus laparoscopic and robotic versus open comparison.

In fact, excluding hybrid procedures in both main comparisons, and organizing the studies according to Ivor-Lewis or McKeown procedures, we could confirm the superiority of robotic approach.

Despite these results, major limitation of this study has to be addressed. As known, meta-analysis has to be considered the mirror of the current literature and, thus, the major limitation of our report is that most studies are on a retrospective manner, foreclosing the possibility to exclude patients selection bias.

We cannot exclude that patients’ allocation into robotic, laparoscopic or open group would be related to surgeons’ preference and experience, patients’ and tumors’ characteristics.

## 11. Conclusions

Even if further randomized clinical trials are needed to give definitive conclusions to include the robotic esophagectomy as the gold standard treatment for esophageal cancer, we can assess that robotic surgery could be considered associated with several advantages over both open and laparoscopic surgery.

Take home messages from our analysis are:

- robotic surgery could be considered absolutely safe, being the results about postoperative complications comparable to open and laparoscopic surgery;
- robotic surgery could be considered superior to open approach, being guaranteed less postoperative complications and superior oncologic results;
- robotic approach appeared to be slightly superor to laparoscopic surgery, providing less postoperative pneumonia and higher number of harvested nodes;
- being by our results safety and effectiveness of robotic surgery to treat esophageal cancer, future perspective is the call to perform randomized clinical trial to confirm the advantages of robotic surgery. Definitive conclusions cannot be drawn, due to limitations of the current literature.

## Figures and Tables

**Table 1:** Characteristics of the included studies comparing robotic and laparoscopic approach.

Study	Study Design	N. of Enrolled Patients		Mean Age	Mean BMI	ASA Score (%)				Tumor Stage (%)					Tumor Localization (%)			
		RAMIE	MIE			I	II	III	IV	0	I	II	III	IV				
Ali et al., 2020	retro	1543	5118	63.71	NR	NR	NR	NR	NR	NR	NR	NR	NR	NR	NR	NR	NR	NR
Chao et al., 2018	retro	34	34	55.12	NR	NR	NR	NR	NR	0.00	47.10	0.00	52.90	0.00	29.40	50.00	20.60	0.00
Chao et al., 2020	retro	39	67	55.60	22.34	NR	NR	NR	NR	0.00	0.00	7.55	92.45	0.00	22.65	48.08	29.27	0.00
Chen et al., 2019	retro	54	54	61.80	22.85	NR	NR	NR	NR	NR	NR	NR	NR	NR	NR	NR	NR	NR
Deng et al., 2018	prosp	52	52	60.95	NR	NR	NR	NR	NR	0.00	12.50	45.15	37.50	0.00	16.35	60.60	22.10	0.95
Duan et al., 2020	retro	109	75	60.45	NR	NR	NR	NR	NR	NR	NR	NR	NR	NR	NR	NR	NR	NR
Gong et al., 2020	retro	91	144	NR	NR	NR	NR	NR	NR	0.00	18.59	33.33	20.83	2.57	3.53	33.01	38.78	0.00
Grimminger et al., 2019	prosp	25	25	62.05	25.55	NR	NR	NR	NR	0.00	0.00	0.00	0.00	0.00	0.00	14.00	89.00	0.00
Harbison et al., 2019	retro	100	625	64.00	27.63	17.38	0.00	77.66	4.97	NR	NR	NR	NR	NR	NR	NR	NR	NR
He et al., 2018	retro	27	27	61.30	21.70	NR	NR	NR	NR	0.00	0.00	0.00	0.00	0.00	7.40	61.10	31.45	0.00
Meredith et al., 2020	prosp	144	95	50.97	21.84	0.35	35.94	42.15	0.67	0.00	15.70	25.11	35.37	2.22	NR	NR	NR	NR
Motoyama et al., 2019	retro	21	38	64.10	NR	NR	NR	NR	NR	0.00	38.80	15.14	45.71	0.00	25.78	38.80	35.42	0.00
Park et al., 2016	retro	62	43	65.08	23.42	30.50	65.72	3.84	0.00	0.00	58.08	25.79	15.24	0.94	14.29	22.85	62.86	0.00
Shirakawa et al., 2020	retro	51	51	68.00	21.95	21.60	64.70	13.70	NR	NR	NR	NR	NR	NR	18.60	46.10	25.50	3.90
Suda et al., 2012	prosp	16	20	65.39	20.78	NR	NR	NR	NR	2.78	33.33	11.11	50.00	2.78	11.11	52.78	36.11	0.00
Tagkalos et al., 2020	prosp	40	40	62.50	26.00	NR	NR	NR	NR	NR	NR	NR	NR	NR	NR	NR	NR	NR
Tsunoda et al., 2021	retro	45	45	NR	NR	9.00	89.00	2.00	0.00	0.00	51.00	19.00	23.00	7.00	14.50	28.00	57.50	0.00
Weksler et al., 2012	retro	11	26	62.64	27.66	NR	NR	NR	NR	27.02	32.39	16.23	24.35	0.00	NR	NR	NR	NR
Weksler et al., 2017	retro	569	569	41.90	NR	NR	NR	NR	NR	4.70	22.73	19.33	17.53	2.40	NR	NR	NR	NR
Xu et al., 2020	retro	292	292	64.63	23.09	17.50	76.05	6.50	0.00	0.00	38.90	20.90	39.05	1.15	7.50	73.30	19.20	0.00
Yang et al., 2020	retro	271	271	63.45	23.20	1.50	89.50	9.05	0.00	0.00	28.20	33.75	26.95	11.05	12.70	62.75	24.55	0.00
Yerokun et al., 2016	retro	170	170	62.95	NR	NR	NR	NR	NR	0.00	0.00	0.00	0.00	0.00	2.95	51.15	45.90	0.00
Zhang et al., 2019	retro	66	66	62.15	23.00	42.45	52.25	5.30	0.00	6.10	27.25	43.20	23.45	0.00	0.00	21.95	28.05	0.00

BMI: Body Mass Index; RAMIE: Robot-Assisted Minimally Invasive Esophagectomy; MIE: Minimally Invasive Esophagectomy; RCT: Randomized Controlled Trial; NR: not reported.All studies reported on the surgical approach, adopting totally robotic or totally laparoscopic approach, except for the study by Yerokun et al. [55] and Harbison et al. [33], in which a hybrid approach was used and for the study by Weksler et al. [52], in which information on surgical procedures was insufficiently provided.

**Table 2:** Characteristics of the included studies comparing robotic and open approach.

Study	Study Design	N. of Enrolled Patients		Mean Age	Mean BMI	ASA Score (%)				Tumor Stage (%)					Tumor Localization (%)			
		RAMIE	OPEN			I	II	III	IV	0	I	II	III	IV				
Espinoza-Mercado et al., 2019	retro	406	406	64	NR	NR	NR	NR	NR	14.65	25.75	37.8	21.3	0	NR	NR	NR	NR
Gong et al., 2020	retro	91	77	NR	NR	NR	NR	NR	NR	2.974	33.33	39.88	21.42	2.38	26.18	37.50	31.54	0.91
Jeong et al., 2016	retro	88	159	NR	22.66	NR	NR	NR	NR	41.19838	42.53	13.12	2.78	0.35	NR	NR	NR	NR
Mehdorn et al., 2020	prosp	11	11	63.8	27.4	0	31.85	68.15	0	13.65	9.1	36.4	27.3	4.55	NR	NR	NR	NR
Meredith et al., 2020	prosp	144	475	64.46	28	0.38	49.62	49.20	0.70	9.66	34.78	41.24	12.27	44.43	NR	NR	NR	NR
Osaka et al., 2018	retro	30	30	62.5	NR	NR	NR	NR	NR	26.65	45	18.3	10	0	26.65	48.35	26.35	0
Pointer et al., 2020	retro	222	222	NR	NR	NR	NR	NR	NR	NR	NR	NR	NR	NR	NR	NR	NR	NR
Rolff et al., 2017	retro	56	160	64.65	26.51	27.03	50	23.22	0.51	NR	NR	NR	NR	NR	NR	NR	NR	NR
Sarkaria et al., 2019	prosp	64	106	61.89	29.12	0	14.16	79.38	6.48	22.48	32.52	24.83	8.24	0	0	1.18	63.54	41.54
Weksler et al., 2017	retro	569	569	63	NR	NR	NR	NR	NR	20.4	32.15	27.7	14.35	35.55	NR	NR	NR	NR
Yun et al., 2020	prosp	130	241	62.92	23.21	NR	NR	NR	NR	20.74	19.16	20.51	3.78	0	43.62	31.80	5.92	4.54

BMI: Body Mass Index; RAMIE: Robot-Assisted Minimally Invasive Esophagectomy; RCT: Randomized Controlled Trial; NR: not reported.

## Data Availability

Not applicable.

## Supplementary Materials

The following are available online at https://www.mdpi.com/article/10.3390/jpm11070640/s1, Supplementary Figure S1. Funnel plot analysis of the comparison between robotic and laparoscopic surgery about intraoperative outcomes. (a) operative time; (b) estimated blood loss; (c) conversion. Supplementary Figure S2. Funnel plot analysis of the comparison between robotic and laparoscopic surgery about postoperative complications. (a) anastomotic leakage; (b) postoperative bleeding; (c) wound infection; (d) pneumonia; (e) RLN paralysis; (f) chylothorax; (g) mortality. Supplementary Figure S3. Funnel plot analysis of the comparison between robotic and laparoscopic surgery about oncologic outcomes. (a) number of harvested nodes; (b) R0 resection. Supplementary Figure S4. Funnel plot analysis of the comparison between robotic and laparoscopic surgery about length of hospital stay. Supplementary Figure S5. Funnel plot analysis of the comparison between robotic and laparoscopic surgery about long-term outcomes. (a) recurrences; (b) 5-years overall survival. Supplementary Figure S6. Funnel plot analysis of the comparison between robotic and open surgery about intraoperative outcomes. (a) operative time; (b) estimated blood loss. Supplementary Figure S7. Funnel plot analysis of the comparison between robotic and open surgery about postoperative complications. (a) anastomotic leakage; (b) postoperative bleeding; (c) wound infection; (d) pneumonia; (e) RLN paralysis; (f) chylothorax; (g) re-operation rate; (h) mortality. Supplementary Figure S8. Funnel plot analysis of the comparison between robotic and open surgery about oncologic outcomes. (a) number of harvested nodes; (b) R0 resection. Supplementary Figure S9. Funnel plot analysis of the comparison between robotic and open surgery about length of hospital stay. Supplementary Figure S10. Funnel plot analysis of the comparison between robotic and open surgery about 5-years overall survival. Supplementary Table S1. NOS quality assessment of the included studies comparing robotic and laparoscopic approach. Supplementary Table S2. NOS quality assessment of the included studies comparing robotic and open approach. Supplementary Table S3. Comparison between robotic and open surgery in terms of pneumonia rate.
