# Supplementary material for: Robotic Esophagectomy. A Systematic Review with Meta-Analysis of Clinical Outcomes
Source: J Pers Med. 2021 Jul 6;11(7):640. doi: 10.3390/jpm11070640 (PMC8306060; doi:10.3390/jpm11070640)
Supplement: Supplementary file 1 [file jpm-11-00640-s001.zip › jpm-1217841-supplementary.pdf]

	Robotic		Open	
AUTHORS	*Harvested nodes (means ±SD)*	*Patients*	*Harvested nodes (means ±SD)*	*Patients*
Espinoza-Mercado et al. 2019	17 ±2.2	406	13 ±2.4	406
Gong et al. 2020	22.84 ±8.37	91	24.09 ±10.77	77
Mehdorn et al. 2020	29.9 ±9.8	11	18.1 ±13.8	11
Meredith et al. 2020	20 ±9	144	10 ±6	475
Osaka et al. 2018	26.9 ±12.2	30	23.6 ±6.6	30
Rolff et al. 2017	29.6 ±10	56	24 ±9.2	160
Sarkaria et al. 2019	26.5 ±9	64	22.3 ±9.9	106
van der Sluis et al. 2019	26.7 ±3.5	54	24.8 ±3	55
Weksler et al. 2017	16 ±2.1	569	13 ±2.1	569
Yun et al. 2020	39.1 ±13.8	130	38.3 ±12.9	241

SD: Standard Deviation
